# The Annoyances of the Administration of Chloroform

**Published:** 1861-05

**Authors:** 


THE ANNOYANCES OF THE ADMINISTRATION OF CHLOROFORM.—There is continually appearing evidence of the loss of confidence in chloroform. The eminent French surgeon, Robert, who seems to ignore ether, in the recent volume of his lectures, makes the following remarks in regard to the administration of chloroform:

"The first condition, for the performance with safety of a long and delicate operation, is that the surgeon should have his mind quite free. Now, however intelligent the assistants who manage the inhalation may be, still the administration of chloroform is a great pre-occupation for the surgeon. I confess that, for my part, I would rather in such a case not perform the operation than do it with constant fear of seeing some accident occur. So that you must reason with the patient and persuade him to allow the operation to be done without chloroform, and exaggerate to him the dangers of anæsthesia."—*Medical and Surgical Reporter.*

